# Flagellum Is Responsible for Promoting Effects of Viable *Escherichia coli* on Calcium Oxalate Crystallization, Crystal Growth, and Crystal Aggregation

**DOI:** 10.3389/fmicb.2019.02507

**Published:** 2019-11-05

**Authors:** Rattiyaporn Kanlaya, Orapan Naruepantawart, Visith Thongboonkerd

**Affiliations:** ^1^Medical Proteomics Unit, Office for Research and Development, Faculty of Medicine Siriraj Hospital, Mahidol University, Bangkok, Thailand; ^2^Immunology Graduate Program, Department of Immunology, Faculty of Medicine Siriraj Hospital, Mahidol University, Bangkok, Thailand

**Keywords:** bacteria, CaOx, crystal, modulator, infection, nephrolithiasis, nidus, promoters

## Abstract

Urease-producing bacteria (especially *Proteus mirabilis*) can cause infection kidney stone. However, recent studies have shown that intact viable non-urease-producing bacteria such as *Escherichia coli* might also promote calcium oxalate (CaOx) kidney stone formation but with unclear mechanism. We thus hypothesized that some relevant bacterial components might be responsible for such promoting effects of the intact viable *E. coli*. Flagella, capsule, lipopolysaccharide (LPS), and outer membrane vesicles (OMVs) were isolated/purified and their stone modulatory activities were evaluated using CaOx crystallization, crystal growth, and crystal aggregation assays. Among these, flagella had the most potent promoting effects on CaOx crystallization, crystal growth, and crystal aggregation. Validation was performed by deflagellation demonstrating that the deflagellated intact viable *E. coli* had markedly reduced CaOx crystal modulatory activities in all aspects (comparable to those of the negative controls). Similarly, neutralization of the isolated/purified flagella using a specific anti-flagellin antibody, not an isotype control, could abolish the promoting effects of flagella. These findings provide direct evidence indicating that flagellum is responsible for the promoting effects of the viable *E. coli* on CaOx crystallization, crystal growth and aggregation.

## Introduction

Urinary tract infection (UTI) is frequently associated with kidney stone disease with two possible dilemmas (Frang et al., 1981; Miano et al., 2007). First, UTI is the priming event leading to kidney stone formation and the stone generated following UTI is termed “infection stone,” which has been previously referred only to infection caused by urease-producing bacteria, especially *Proteus mirabilis* (Bichler et al., 2002; Miano et al., 2007; Flannigan et al., 2014). The most common infection stone type is struvite, which is composed mainly of magnesium ammonium phosphate (Bichler et al., 2002; Miano et al., 2007; Flannigan et al., 2014). For the second dilemma, UTI on the other hand is a complication following “metabolic stone” [e.g., calcium oxalate (CaOx), calcium phosphate, uric acid, etc.], which is primarily caused by metabolic derangement (e.g., hyperoxaluria, hypercalciuria, hyperuricosuria, hypocitraturia, etc.) (Coe et al., 2005; Penniston et al., 2007; Richman et al., 2014). However, recent *in vivo* evidence has suggested that some common non-urease producing bacteria such as *Escherichia coli* might also induce formation of CaOx stone, the most common type of previously classified “metabolic stone” (Tavichakorntrakool et al., 2012). Moreover, an *in vitro* study also confirmed that the intact viable *E. coli*, not the dead intact or fragmented *E. coli*, could promote CaOx crystal growth and aggregation and thus might enhance CaOx kidney stone formation processes (Chutipongtanate et al., 2013). However, mechanism underlying the promoting effects of the intact viable *E. coli* on CaOx stone formation remained unclear. We thus hypothesized that some bacterial components or organelles might be responsible for such promoting activities of the intact viable *E. coli* on CaOx stone formation. Flagella, capsule, lipopolysaccharide (LPS), and outer membrane vesicles (OMVs) were isolated/purified and their stone modulatory activities were evaluated using CaOx crystallization, crystal growth, and crystal aggregation assays.

## Materials and Methods

### Bacterial Culture

Single colony of *E. coli* ATTC 25922 (ATCC; Manassas, VA, United States) was inoculated into 5 ml LB broth (1% tryptone, 1% yeast extract and 1% NaCl) (Becton Dickinson; Sparks, MD, United States) and incubated in a shaking incubator at 37°C for 16 h until the absorbance or optical density at λ600 nm was 0.955 (at which approximately 5 × 10^6^ colony forming unit (CFU)/ml was achieved). Thereafter, 1 ml of the bacterial starter was inoculated into 100 ml of fresh LB broth and grown in a shaking incubator at 37°C for 3 h to reach its mid-log phase.

### Isolation of Flagellum and Confirmation

Flagellar isolation was performed using pH shock method as described previously (Craige et al., 2013). Briefly, 100 ml of mid-log- phase bacteria was centrifuged at 1,500 × *g* for 5 min and the bacterial pellet was washed and resuspended in 10 ml of 10 mM HEPES [4-(2-hydroxyethyl)-1-piperazineethanesulfonic acid] (Sigma-Aldrich; St. Louis, MO, United States). The pH was acidified to 4.5 by incubating with 0.5 N acetic acid (RCI Labscan; Bangkok, Thailand) for 45 sec and then neutralized to 7.0 using 0.5 M KOH (AppliChem GmbH; Darmstadt, Germany). The bacterial suspension was centrifuged at 10,000 × *g* for 30 min to remove bacterial cells. A supernatant containing flagella was centrifuged at 100,000 × *g* for 1 h. The flagellar pellet was then resuspended in a basic buffer (10 mM Tris–HCl and 90 mM NaCl; pH 7.4).

Confirmation of flagellar isolation was done by morphological examination using Gray’s method (Gray, 1926). Briefly, the isolated flagella were smeared on a glass slide and iron tannate dye (Sigma-Aldrich) was dropped onto the glass slide, incubated at 25°C for 10 min and rinsed with distilled water. The glass slide was further flooded with carbol-fuchsin (Sigma-Aldrich) for 10 min, rinsed with tap water, and then air dried before examining under a light microscope.

### Isolation of Capsule and Confirmation

Capsule isolation was performed using the protocol described previously (Lu et al., 2008) with slight modifications. Briefly, 100 ml of mid-log- phase bacteria was centrifuged at 1,500 × *g* for 5 min and the bacterial pellet was resuspended in 25 ml PBS. The bacterial suspension was sonicated and precipitated by ice-cold acetone (Fisher Scientific; Loughborough, United Kingdom). The capsular polysaccharide (exopolysaccharide) pellet was then collected by a centrifugation at 6,000 × *g* for 10 min and then resuspended in distilled water. The crude exopolysaccharide was dialyzed against large volume of distilled water, concentrated by lyophilization, and then dissolved in 10 mM MgCl_2_. Deoxyribonuclease I (DNase I) (New England Biolabs; Ipswich, MA, United States) and ribonuclease A (RNase A) (Invitrogen; Paisley, United Kingdom) were added to final concentrations of 5 μg/ml and 0.1 mg/ml, respectively, and incubated at 37°C in a shaking water bath for 5 h. Trypsin (Gibco; Grand Island, NY, United States) was added to a final concentration of 0.1 mg/ml and further incubated at 37°C in a shaking water bath for 24 h. Thereafter, the mixture was heated at 80°C for 30 min and centrifuged at 10,000 × *g* for 5 min, and the supernatant was dialyzed and lyophilized. A powder of crude exopolysaccharide was dissolved in 50 mM Tris-base (pH 8) added with 1.5 mM sodium deoxycholate (Sigma-Aldrich). The mixture was further incubated at 65°C for 15 min, chilled on ice for 15 min, and then added with 20% acetic acid to a final concentration of 1%. Contaminants were pelleted off by centrifugation at 10,000 × *g* for 5 min, whereas the supernatant containing isolated capsules was collected, dialyzed and lyophilized. Finally, the isolated capsules were resuspended in the basic buffer.

Confirmation of the isolated capsules was done by detecting total carbohydrate content using phenol-sulfuric acid method as described previously (BeMiller, 2017) with slight modifications. Briefly, 2 μl of the sample was added into a well containing 30 μl distilled water. After adding 150 μl H_2_SO_4_, the mixture was incubated at 90°C for 15 min before adding with 30 μl of 5% phenol. Finally, the absorbance of the mixture was measured at λ490 nm. BSA (1 mg/ml) and glucose (1 mg/ml) were used as negative and positive controls, respectively.

### Isolation of LPS and Confirmation

LPS was isolated by continuing the processes in parallel with capsular isolation. After crude exopolysaccharide was precipitated with 20% acetic acid, LPS and deoxycholate were pelleted off by centrifugation at 10,000 × *g* for 5 min. The pellet was then resuspended in distilled water and further dialyzed against distilled water to eliminate deoxycholate. Thereafter, the LPS sample was concentrated by lyophilization.

Confirmation of the LPS isolation was done by electrophoretic method. Briefly, the LPS sample was resuspended in Laemmli buffer, heated and then resolved by SDS-PAGE using 12% polyacrylamide gel and visualized by silver staining to observe the ladder-like pattern.

### Isolation of OMVs and Confirmation

Isolation of OMVs was performed according to the previous study (Lee et al., 2007) with slight modifications. Briefly, 100 ml of mid-log- phase bacteria was centrifuged at 1,500 × *g* for 5 min and the supernatant was collected and filtered by using a 0.2-μm membrane filter (Sartorius Stedim Biotech; Goettingen, Germany). To collect OMVs, the flowthrough supernatant was centrifuged at 150,000 × *g* and 4°C for 1 h using an ultracentrifuge (Sorvall; Langenselbold, Germany).

Confirmation of the OMVs isolation was done by Western blotting for the OMVs marker using the whole cell bacteria as the positive control. Separation was done by 12% SDS-PAGE at 150V for 2 h and the resolved proteins were electrotransferred onto a nitrocellulose membrane using a semi-dry transfer apparatus (GE Healthcare; Uppsala, Sweden) at 85 mA for 1.5 h. Non-specific bindings were blocked with 5% skim milk in PBS at 25°C for 1 h. The membrane was then probed with a mouse monoclonal anti-GroEL antibody (Santa Cruz Biotechnology; Santa Cruz, CA, United States) (diluted 1:1,000 in 1% skim milk/PBS) at 4°C overnight. After washing with PBS three times, the membrane was incubated with corresponding secondary antibody conjugated with horseradish peroxidase (1:2,000 in 1% skim milk/PBS) (Dako; Glostrup, Denmark) at 25°C for 1 h. Immunoreactive band was developed by SuperSignal West Pico chemiluminescence substrate (Pierce Biotechnology; Rockford, IL, United States) and were then visualized by autoradiogram.

### Preparation of Intact Viable and Intact Dead Bacteria

Intact viable bacteria and intact dead bacteria were used as positive and negative controls, respectively, in all assays. Bacterial concentration was adjusted to 1 × 10^5^CFU/ml for each assay. Intact dead bacteria were prepared as previously reported (Chutipongtanate et al., 2013). Briefly, 1 × 10^5^CFU/ml bacteria were added with 4% formaldehyde in PBS and incubated at 25°C for 30 min. Thereafter, the bacterial pellet was collected and washed twice with PBS. Plate counting assay was then performed to ensure that all the bacteria were dead and there were no viable bacteria left after formaldehyde treatment. Briefly, a bacterial suspension was 10-fold serially diluted in LB broth and 100 μl of each dilution was spread onto an LB agar plate and incubated at 37°C for 12–16 h. The bacterial colonies were counted and calculated as CFU/ml. In addition, flagellar staining was performed using Gray’s method (as aforementioned) to examine the presence of flagella in the intact viable and dead bacteria.

### Preparation of Deflagellated Bacteria

Deflagellated bacteria were prepared by the pH shock method similar to the method described above for flagellar isolation. Briefly, 100 ml of mid-log- phase bacteria was centrifuged at 1,500 × *g* for 5 min and the bacterial pellet was washed and resuspended in 10 ml of 10 mM HEPES. The pH was acidified to 4.5 by incubating with 0.5 N acetic acid for 45 sec and then neutralized to 7.0 using 0.5 M KOH. The bacterial suspension was added with 1 mg/ml chloramphenicol to prevent regeneration of flagella and centrifuged at 10,000 × *g* for 30 min to collect the deflagellated bacterial cells. The viability of the deflagellated bacteria was evaluated by plate counting assay as described above.

### Neutralization of the Flagellar Effects Using a Specific Anti-flagellin Antibody

To neutralize the promoting effects of flagella on CaOx crystallization, crystal growth and aggregation, the isolated/purified flagella were incubated with 2 μg mouse monoclonal anti-flagellin antibody (BioLegend, Inc., San Diego, CA, United States) in the basic buffer at 25°C for 1 h prior to CaOx crystallization, crystal growth and crystal aggregation assays as described below. An equal amount of the isotype IgG (Santa Cruz Biotechnology, TX, United States) was used as the control for this neutralization assay.

### CaOx Crystallization Assay

CaOx crystallization assay was performed as previously described (Amimanan et al., 2017; Manissorn et al., 2017). Briefly, each bacterial component was derived from approximately 4 × 10^7^ bacteria, resuspended in 4 μl basic buffer, and then added into each well of a 24 well-plate (Corning Inc., Corning, NY, United States) containing 500 μl of 10 mM CaCl_2_ in the basic buffer. Thereafter, 500 μl of 1 mM Na_2_C_2_O_4_ in the basic buffer was added and incubated at 25°C for 60 min. Crystal images were captured randomly from at least 15 high-power fields (HPFs) under Nikon Eclipse Ti-S inverted phase-contrast light microscope (Nikon; Tokyo, Japan). Crystal size and number were quantitated using NIS Element D software version 4.11 (Nikon) from at least 100 crystals from 15 HPFs in each biological replicate. Crystal mass was then calculated using the following formula:

Formula 1: Crystal mass (μm^2^/HPF) = Crystal size (μm^2^) × Number of crystals (/HPF)

### CaOx Crystal Growth Assay

CaOx crystal growth assay was performed as previously described (Thongboonkerd et al., 2008; Peerapen and Thongboonkerd, 2016). Briefly, CaOx crystal seeds were generated by adding 500 μl of 1 mM Na_2_C_2_O_4_ (in the basic buffer) into each well of a 24 well-plate containing 500 μl of 10 mM CaCl_2_ (in the basic buffer) and incubated at 25°C for 60 min. Thereafter, each bacterial component derived from approximately 4 × 10^7^ bacteria and finally resuspended in 4 μl basic buffer was added into each well and this time-point was defined as T_0_. The mixture was further incubated for 60 min (T_60_). At T_0_ and T_60_, crystal images were captured randomly from at least 15 HPFs under Nikon Eclipse Ti-S inverted phase-contrast light microscope (Nikon). Crystal sizes at T_0_ and T_60_ were measured using NIS Element D software version 4.11 (Nikon), whereas crystal growth (represented by Δ Crystal size) was calculated from at least 100 crystals in 15 HPFs in each biological replicate using the following formulas:

Formula 2: Δ Crystal size (μm^2^) = Crystal size at T_60_ − Crystal size at T_0_
Formula 3:% Crystal growth promotion = (T-B)/B × 100

(Where T was the Δ Crystal size of the tested sample and B was the Δ Crystal size of the blank control.)

### CaOx Crystal Aggregation Assay

CaOx crystal aggregation assay was performed as previously described (Chaiyarit and Thongboonkerd, 2017; Khamchun et al., 2019). Briefly, individual CaOx crystals were generated as aforementioned but with a larger volume in a 50-ml conical tube (Corning Inc., NY, United States) and then harvested by centrifugation at 2,000 × *g* for 5 min. The supernatant was discarded, whereas CaOx crystals were washed three times with methanol. After another centrifugation at 2,000 × *g* for 5 min, methanol was discarded and the crystals were air-dried overnight at 25°C. CaOx crystals (1,000 μg dry weight) were resuspended in 1 ml of the basic buffer in each well of the 6-well plate (Corning Inc., NY, United States). Thereafter, each bacterial component derived from approximately 4 × 10^7^ bacteria and finally resuspended in 4 μl basic buffer was added into each well and then incubated in a shaking incubator at 150 rpm and 25°C for 1 h. Thereafter, formation of CaOx crystal aggregate (defined as an assembly of three or more individual COM crystals that tightly joined together) was observed and imaged under Nikon Eclipse Ti-S inverted phase-contrast light microscope (Nikon). Number of COM crystal aggregates was counted from at least 15 randomized HPFs in each biological replicate.

### Statistical Analysis

All of the aforementioned experiments were done in triplicate (three independent experiments) and the quantitative data are reported as mean ± SEM. Differences between two independents groups was analyzed by Mann-Whitney *U* test, whereas multiple comparisons were performed using Kruskal Wallis test. *P* value less than 0.05 was considered statistically significant.

## Results

### Isolation of Individual Bacterial Components and Confirmation

**FIGURE 1:**
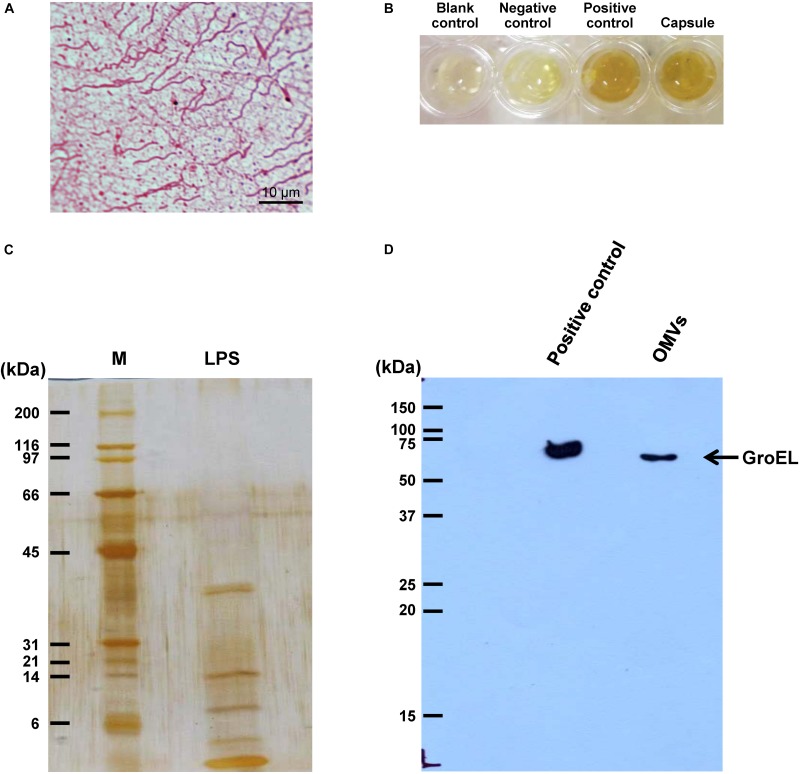
Isolation of individual bacterial components and confirmation. Bacterial components including flagellum, capsule, LPS, and OMVs were isolated from intact viable *Escherichia coli*. **(A)** Typical morphology of flagellum was visualized by Gray’s method. **(B)** Total carbohydrate content of the isolated capsule was validated by phenol-sulfuric acid method. BSA and glucose served as the negative and positive controls, respectively. **(C)** The ladder-like pattern of LPS was visualized by SDS-PAGE and silver staining (M stands for molecular weight standard ladder). **(D)** GroEL, one of the protein markers of bacterial OMVs, was confirmed by Western blot analysis.

Individual bacterial components, including flagellum, capsule, LPS, and OMVs, were isolated and purified from an equal number of *E. coli*. Typical morphology of flagellum with helical hair-like filament and length of approximately 5–10 μm was observed (Figure 1A). Bacterial capsule comprising acidic polysaccharide (classified as carbohydrate) was confirmed by phenol-sulfuric acid method as indicated by the dark yellow color comparable to that of glucose, which served as the positive control (Figure 1B). In addition, the ladder-like pattern of isolated LPS was visualized by silver staining (Figure 1C). Moreover, GroEL (a marker for bacterial OMVs) was clearly observed in the isolated OMVs fraction (Figure 1D). These findings confirmed that each bacterial component was successfully isolated from the whole cell of *E. coli*.

### Effect of Bacterial Components on CaOx Crystallization

**FIGURE 2:**
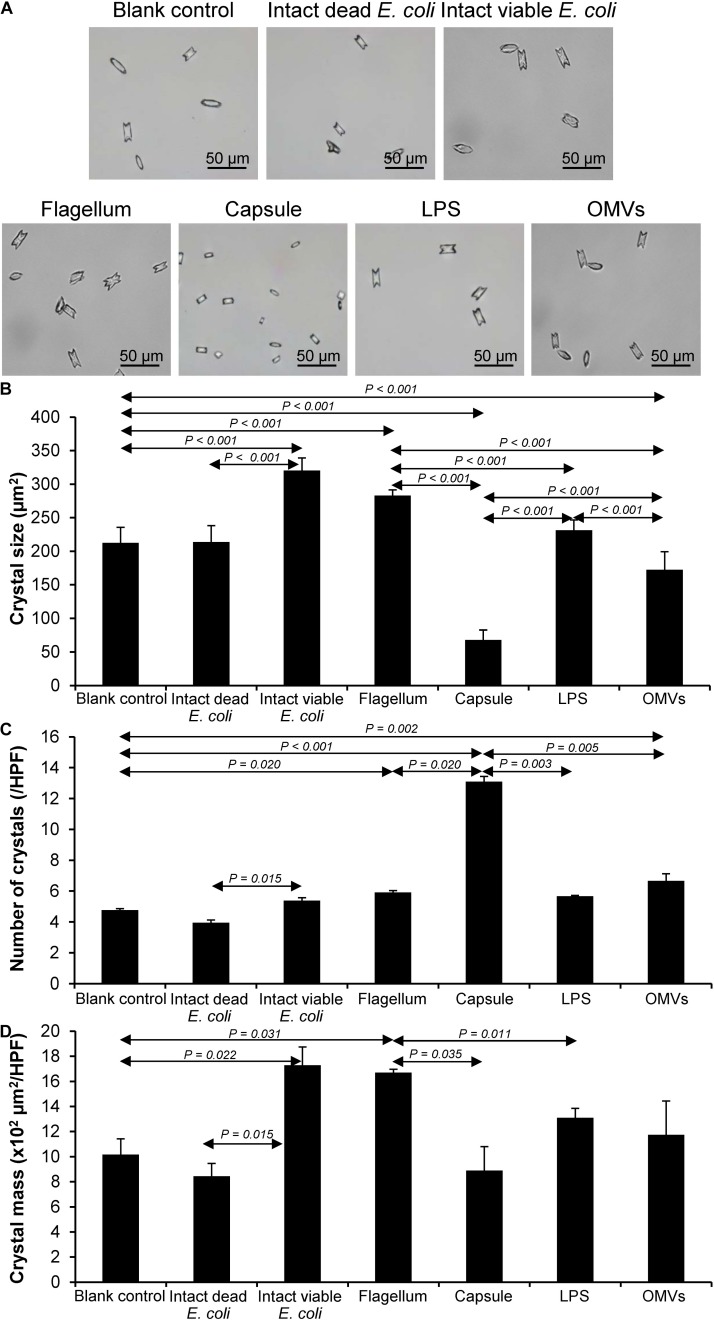
Effect of bacterial components on CaOx crystallization. Crystallization assay was performed in the presence or absence of each bacterial component derived from equal number of bacteria (4 × 10^7^ CFU). Intact viable *E. coli* and intact dead *E. coli* served as the positive and negative controls, respectively. **(A)** Crystal morphology and size in each condition after 60-min crystallization. **(B)** Crystal size. **(C)** Crystal number. **(D)** Crystal mass (see Formula 1 in section “Materials and Methods”). All quantitative data are reported as mean ± SEM derived from three independent experiments.

After being isolated, an equal amount of individual components (including flagellum, capsule, LPS, and OMVs) was tested for their modulatory effects on CaOx crystallization. After 60-min incubation, degree of CaOx crystallization was evaluated by measuring crystal size. As expected, intact viable *E. coli* (which served as the positive control) obviously enhanced crystallization, while intact dead bacteria (which served as the negative control) had no effect on crystallization (comparable to the blank control) (Figure 2). The data on bacterial components showed that flagella significantly increased crystal size (Figure 2B), whereas capsule, OMVs and flagella significantly increased the crystal number (Figure 2C). For crystal mass, which considered both size and number of the crystals (see section Formula 1 in “Materials and Methods”), only flagella significantly promoted crystallization (Figure 2D). These data indicated that flagella had the most potent promoting effect on CaOx crystallization.

### Effect of Bacterial Components on CaOx Crystal Growth

**FIGURE 3:**
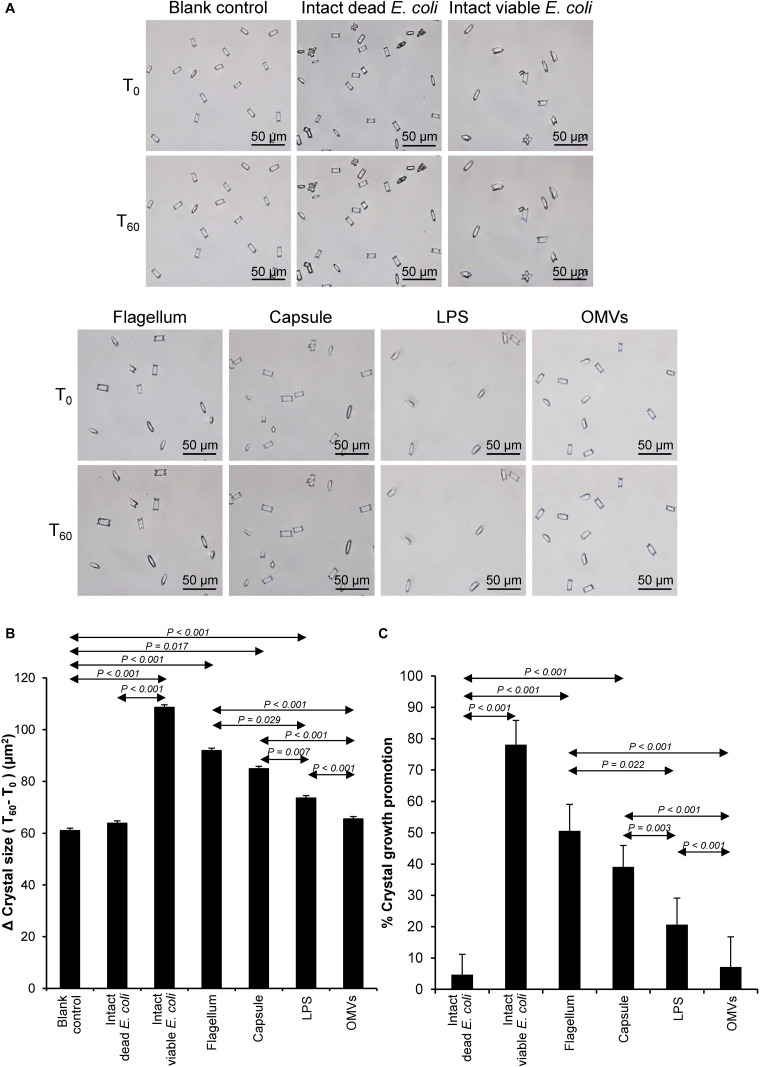
Effect of bacterial components on CaOx crystal growth. Crystal growth assay was performed in the presence or absence of each bacterial component derived from equal number of bacteria (4 × 10^7^ CFU). Intact viable *E. coli* and intact dead *E. coli* served as the positive and negative controls, respectively. **(A)** Crystal morphology and size in each condition at T_0_ and T_60_. **(B)** Δ Crystal size (difference in crystal size between T_0_ and T_60_) was analyzed. **(C)** % Crystal growth promotion was calculated. See calculating formulas in section “Materials and Methods.” Quantitative data are reported as mean ± SEM derived from three independent experiments.

Crystal growth was evaluated by measuring Δ Crystal size and% Crystal growth promotion after allowing the crystal seeds to grow for 60 min. The intact dead and intact viable bacteria served as the negative and positive controls, respectively. The data showed that flagella and capsule exhibited promoting effect on CaOx crystal growth as indicated by the significant increases in Δ Crystal size and% Crystal growth promotion comparing to the blank control and intact dead *E. coli* (Figure 3). Interestingly, flagella provided the most potent promoting effect on CaOx crystal growth.

### Effect of Bacterial Components on CaOx Crystal Aggregation

**FIGURE 4:**
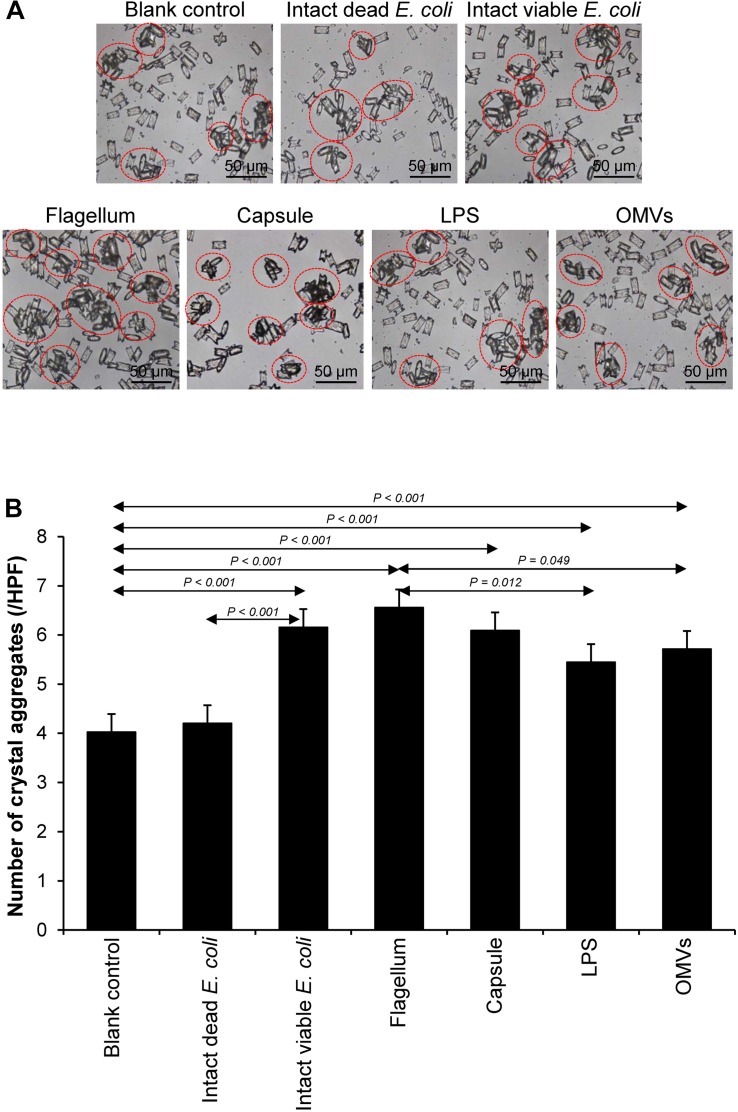
Effect of bacterial components on CaOx crystal aggregation. Crystal aggregation assay was performed in the presence or absence of each bacterial component derived from equal number of bacteria (4 × 10^7^ CFU). Intact viable *E. coli* and intact dead *E. coli* served as the positive and negative controls, respectively. **(A)** Crystal aggregates are indicated by dashed circles. **(B)** Quantitative data of crystal aggregates are reported as mean ± SEM derived from three independent experiments.

Crystal aggregation was evaluated by measuring number of the crystal aggregates, which is one of the best indices to quantitate the degree of CaOx crystal aggregation (Chaiyarit and Thongboonkerd, 2017). The intact dead and intact viable bacteria served as the negative and positive controls, respectively. Comparing to the blank and negative controls, all of the bacterial components significantly promoted CaOx crystal aggregation (Figure 4). Again, flagella tended to have the most potent promoting effect on crystal aggregation among the four bacterial components.

### Validation of Promoting Effects of Flagella on CaOx Crystallization, Crystal Growth, and Aggregation

**FIGURE 5:**
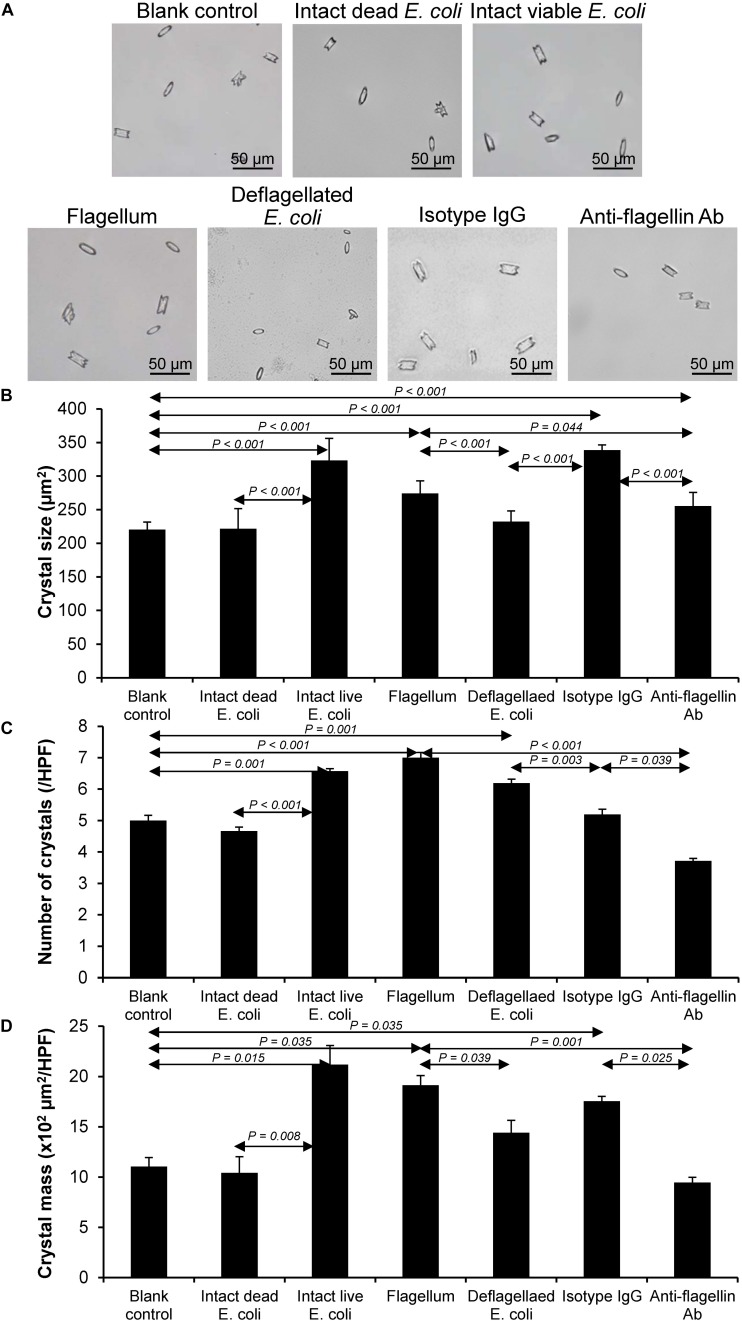
Validation of the promoting effect of flagella on CaOx crystallization. Crystallization assay was performed using intact dead *E. coli* as the negative control and intact viable *E. coli* and flagella as the positive controls. **(A)** Crystal morphology and size in each condition after 60-min crystallization. **(B)** Crystal size. **(C)** Crystal number. **(D)** Crystal mass (see Formula 1 in section “Materials and Methods”). Quantitative data are reported as mean ± SEM derived from three independent experiments.

**FIGURE 6:**
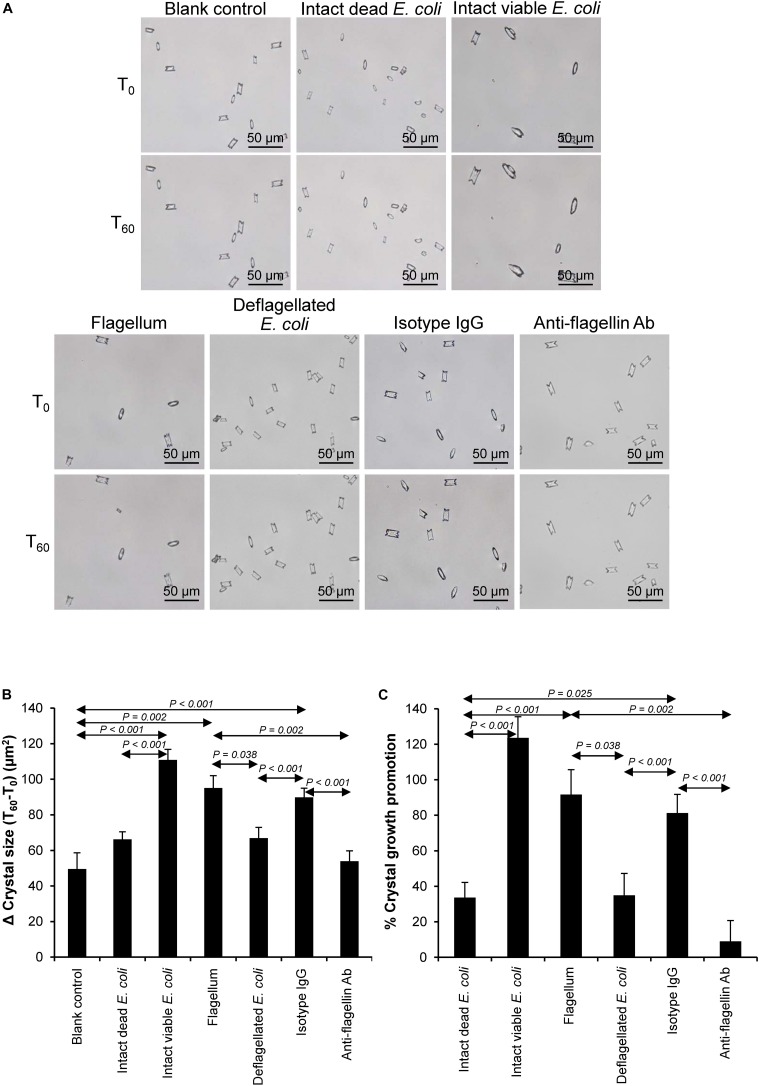
Validation of the promoting effect of flagella on CaOx crystal growth. Crystal growth assay was performed using intact dead *E. coli* as the negative control and intact viable *E. coli* and flagella as the positive controls. **(A)** Crystal morphology and size in each condition at T_0_ and T_60_. **(B)** Δ Crystal size (difference in crystal size between T_0_ and T_60_) was analyzed. **(C)** % Crystal growth promotion was calculated. See calculating formulas in section “Materials and Methods.” Quantitative data are reported as mean ± SEM derived from three independent experiments.

**FIGURE 7:**
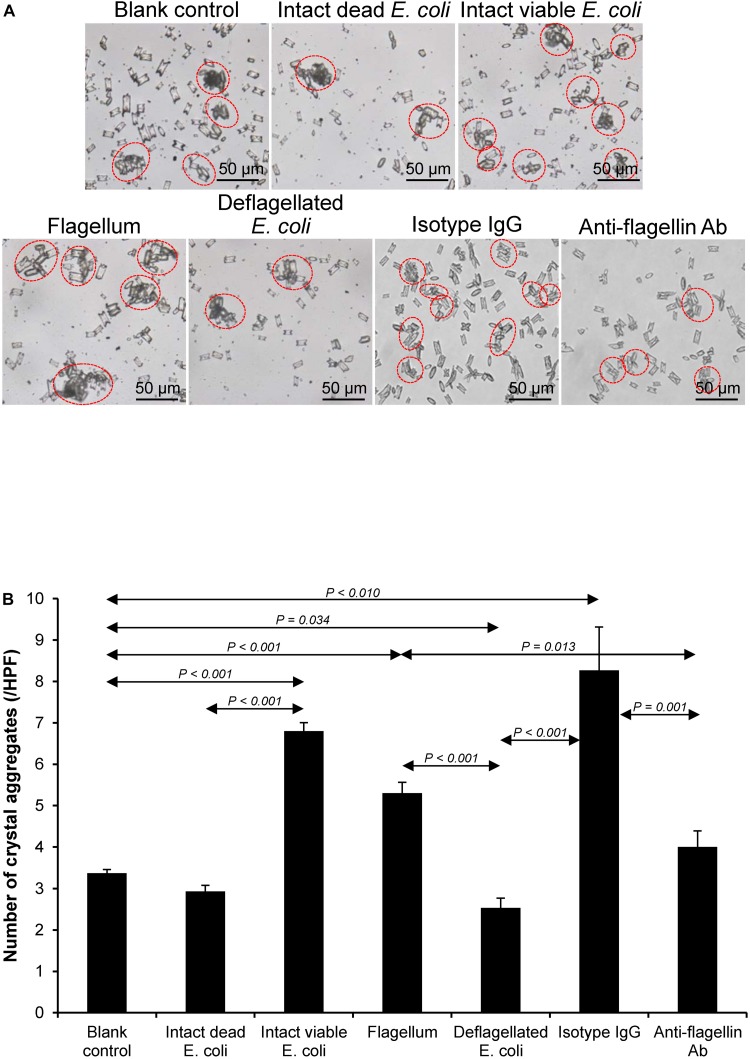
Validation of the promoting effect of flagella on CaOx crystal aggregation. Crystal aggregation assay was performed using intact dead *E. coli* as the negative control and intact viable *E. coli* and flagella as the positive controls. **(A)** Crystal aggregates are indicated by dashed circles. **(B)** Quantitative data of crystal aggregates are reported as mean ± SEM derived from three independent experiments.

**FIGURE 8:**
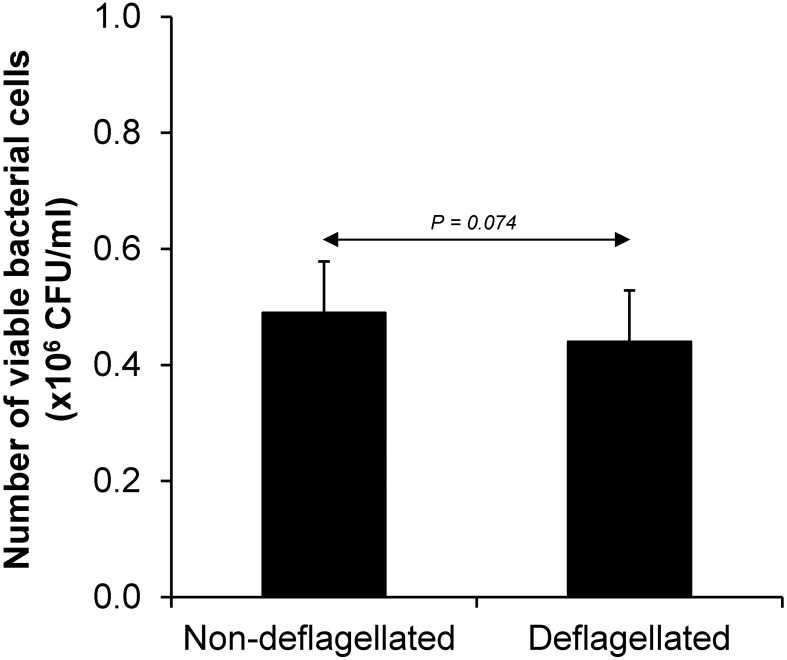
Bacterial viability after flagellar detachment. The intact viable bacteria were subjected to flagellar detachment as detailed in section “Materials and Methods.” An equal number of the non-deflagellated bacteria served as the controls. Both non-deflagellated and deflagellated bacteria were then subjected to viable cell count using a plate counting assay.

Various CaOx crystal assays provided the consistent results, indicating that flagellum was the most potent promoter on crystallization, crystal growth and aggregation (Figures 2–4). To strengthen these findings, the promoting effects of flagella were validated by deflagellation and neutralization with a specific antibody against flagellin (a major component of bacterial flagella). The intact dead bacteria served as the negative control, whereas both the intact viable bacteria and the isolated/purified flagellum served as the positive controls in these validation tests. Interestingly, the results showed that all the promoting effects of flagella were completely abolished in the deflagellated and anti-flagellin-neutralized bacteria, whereas the isotype IgG had no counter activities against the promoting effects of the flagella (Figures 5–7). To further strengthen that flagellum was responsible for the promoting effects of the intact viable *E. coli* on CaOx crystallization, crystal growth and aggregation, flagellar staining was performed. The data demonstrated that flagella were detected only in the intact viable *E. coli*, not in the intact dead *E. coli* (Supplementary Figure S1). Finally, bacterial viability was examined by plate counting assay to ensure that the absence of promoting effects of the deflagellated bacteria on CaOx crystallization, crystal growth and aggregation was not due to a loss of their viability after flagellar detachment. The results revealed no significant difference in bacterial viability between non-deflagellated and deflagellated *E. coli* (Figure 8).

## Discussion

Association between kidney stone formation and bacteria of the family *Enterobacteriaceae* isolated from the stone formers had been previously implicated (Keefe, 1976). Inoculation of different bacterial species (e.g., *Enterobacter aerogenes*, *Proteus vulgaris*, *Edwardsiella tarda*, and *E. coli*, etc.) into a culture medium for up to 7–11 days resulted in generation of different types of crystalline materials, e.g., calcium pyrophosphate, hydroxyl apatite, and calcite-III crystals (Keefe, 1976). Similarly, brushite and hydroxyl apatite were observed when *E. coli* was inoculated in sterilized human urine collected from healthy individuals (Cohen et al., 1982). Without a detailed explanation, these findings suggest that non-urease-producing bacteria might also induce kidney stone formation.

Characterizations of clinical specimens collected from the stone formers in the Northeastern Thailand have unveiled that *E. coli* was accounted for one-third of all bacterial isolates found in both stone nidus and catheterized urine samples (Tavichakorntrakool et al., 2012). In addition, analysis of the stone compositions has shown that the most common type of kidney stones associated with bacterial isolates was CaOx (50%), which had been previously classified as “metabolic stone,” while only 13% accounted for typically “infection stone” (Tavichakorntrakool et al., 2012). Interestingly, stone matrices taken from both nidus and periphery parts contained almost identical bacterial isolates, suggesting that these pathogens were not randomly entrapped into the stones during secondary UTIs but might be associated with the stone formation and disease pathogenesis (Tavichakorntrakool et al., 2012). Collectively, these findings suggest the possible role of non-urease-producing bacteria, in particular *E. coli*, as the causative pathogens for metabolic kidney stone formation. In other words, UTI may not be only the complication that follows stone formation but also an emerging contributor to metabolic stone formation.

In addition, an *in vitro* study has confirmed such hypothesis by showing direct evidence demonstrating that the common uropathogenic bacteria (*E. coli* and *Klebsiella pneumonia*) and non-uropathogenic bacteria (*Staphylococcus aureus* and *Streptococcus pneumoniae*) could significantly enhance CaOx crystal growth and aggregation (Chutipongtanate et al., 2013). Interestingly, such CaOx crystal promoting effects were found only in the intact viable bacteria, but not in the intact dead bacteria or their fragments (Chutipongtanate et al., 2013).

More recent studies have provided increasing evidence of the possible roles of non-urease-producing bacteria in kidney stone formation in addition to their roles in the secondary infection following urological obstruction or urinary stagnation from renal calculi (Barr-Beare et al., 2015; Tavichakorntrakool et al., 2017). The study on interaction between *Enterobacteriaceae* and deposition of CaOx stone in pediatric urolithiasis showed that *E. coli* was identified in a positive stone culture and selectively aggregated on and around CaOx crystals (Barr-Beare et al., 2015). In addition, induction of pyelonephritis by inoculating the uropathogenic *E. coli* in mice with glyoxalate-induced kidney stones resulted in an increase in CaOx crystal deposition, bacterial burden, and kidney inflammatory response when compared to kidney stone-induced mice without bacterial inoculation or mice with pyelonephritis alone (Barr-Beare et al., 2015). These results suggest that bacteria could worsen kidney stone deposition and, more importantly, might be essential for kidney stone formation.

In the present study, the CaOx lithogenic activities of bacterial components, including flagella, capsule, LPS, and OMVs were investigated in order to address mechanisms underlying the promoting effects of intact viable non-urease-producing bacteria on CaOx kidney stone, which was previously classified as “metabolic stone.” *E. coli* was used as a representative for non-urease-producing bacteria because it is the most frequently found organism in the stone nidus and catheterized urine from the CaOx stone formers (Tavichakorntrakool et al., 2012). Our findings revealed that flagella were responsible for the promoting effects of intact viable *E. coli* to promote CaOx stone formation processes, including crystallization, crystal growth, and crystal aggregation (Table 1). For crystallization and growth, a study by Farmanesh et al. (Farmanesh et al., 2014) has revealed that natural modulators of CaOx crystallization found in the stone matrix, including lysozyme and lactoferrin, could promote crystal growth on the basis of colloidal theory as well as the properties of their peptide subdomains. A relative net charge of CaOx crystal in aqueous solution is negative due to a dissociation of calcium ions from the crystal surface (Farmanesh et al., 2014). According to the colloidal theory, some modulators with positive moieties (i.e., peptides or proteins rich with L-arginine and L-lysine residues) can bind with oxalate molecules on the crystal surface and some are prevented by electrostatic repulsion generated by calcium ions in the Stern layer and thus cannot interfere with crystallization and growth (Farmanesh et al., 2014). Our findings showed that flagella exhibited promoting effects on CaOx crystallization and growth (Figures 2, 3). Unlike purified proteins, flagellum is composed of several proteins built into a structural organelle. Therefore, a net charge of flagellum is derived from different pH of proteins and also a microenvironment during test (e.g., pH of the solution, ionic strength, etc.). Alterations in structure during adhesion onto CaOx crystal surface can also affect its dynamic net charge (Clark et al., 1999; Farmanesh et al., 2014). Additionally, the proteins with different pH can modify zeta potential of CaOx crystals from negatively charged to be more positively charged by residing in diffuse double layer explained by the colloidal theory (Farmanesh et al., 2014). We thus assumed that flagella and other bacterial components might promote crystallization and growth of CaOx crystals by this manner.

**TABLE 1:** Summary for the effects of various bacterial components on COM crystals.

**Assay**		**Bacterial component**		
	
	**Flagellum**	**Capsule**	**LPS**	**OMVs**
**Crystallization**				
*Crystal size*	+ + +	−−	+	−
*Crystal number*	+	+ + +	NS	+
*Crystal mass*	+ + +	NS	NS	NS
**Crystal growth**	+ + +	+ +	NS	NS
**Crystal aggregation**	+ + +	+ + +	+ +	+ +

*−, slightly inhibited; −−, moderately inhibited; +, slightly promoted; + +, moderately promoted; + + +, strongly promoted; NS, not significant.*

In addition to its role as a locomotive organelle of the bacteria, flagellum also contributes to biofilm biogenesis and is involved in adherence, maturation, and dispersal (Zhou et al., 2015: Nakamura et al., 2016). A study by Friedlander et al. (2015) has revealed that *E. coli* flagella played role in adhesion to abiotic surfaces, preferentially to the hydrophobic surfaces, not hydrophilic ones. In consistent with these findings, Yoshihara et al. (2015) have demonstrated that an increasing external detachment force was required to remove different strains of *E. coli* from glass substratum and the detachment force order was as follows: wild-type strain with flagella > flagellar paralyzed strain > non-flagellated strain. These results suggest that flagellum and its motility is important for the bacterial adhesion on surfaces (Yoshihara et al., 2015). According to these findings, we speculated that *E. coli* flagella bound to abiotic surfaces of CaOx crystals and then facilitated crystal aggregation.

In this study, OMVs and LPS derived from *E. coli* culture supernatant could promote CaOx crystal growth and aggregation (Figures 3, 4). These results were consistent with the data obtained from a recent study by Amimanan et al. (2017) demonstrating that elongation factor Tu (EF-Tu) on the surface of OMVs of *E. coli* isolated from kidney stone patients could promote CaOx crystal growth and aggregation (Amimanan et al., 2017). A composition analysis of OMVs derived from Gram-negative bacteria including *E. coli* unveiled that the main component of OMVs is LPS with some abundant outer membrane proteins, whereas the inner membrane and cytoplasmic components are highly depleted (Bos et al., 2007; Kulp and Kuehn, 2010). It was thus implicated that the promoting effects of both OMVs and LPS on CaOx crystal growth and aggregation might be due to similar molecular mechanism that might depend on their electrostatic force or zeta potential of the surfaces. This hypothesis is supported by Clark et al. (1999) who previously showed that distribution of electrostatic surface charge of protein could determine the secondary growth of CaOx crystals.

In summary, our findings provided the *in vitro* evidence demonstrating that bacterial components isolated from the intact viable *E. coli*, including flagella, capsule, LPS, and OMVs, promoted CaOx crystallization, crystal growth and aggregation (Table 1), all of which are the important processes required for kidney stone formation (Khan et al., 2016; Bird and Khan, 2017). Among all of these bacterial components, flagella tended to have the most potent promoting effects on CaOx crystallization, crystal growth and aggregation. Deflagellation and neutralization with a specific anti-flagellin antibody could confirm the critical role of flagella on CaOx crystal promoting processes. All of these findings support a hypothesis that non-urease-producing bacteria may also serve as the causative pathogens for CaOx stone, which had been previously classified as “metabolic stone.” Nevertheless, our *in vitro* findings need validation in the *in vivo* settings and also in human studies. Moreover, the roles for other bacteria should be also elucidated.

## Data Availability Statement

All datasets generated for this study are included in the article/Supplementary Material.

## Author Contributions

RK and ON performed the experiments. All authors designed the research, analyzed the data, wrote the manuscript, and reviewed and approved the manuscript.

## Conflict of Interest

The authors declare that the research was conducted in the absence of any commercial or financial relationships that could be construed as a potential conflict of interest.
