# Supplementary material for: Flagellum Is Responsible for Promoting Effects of Viable Escherichia coli on Calcium Oxalate Crystallization, Crystal Growth, and Crystal Aggregation
Source: Front Microbiol. 2019 Nov 5;10:2507. doi: 10.3389/fmicb.2019.02507 (PMC6848068; doi:10.3389/fmicb.2019.02507)
Supplement: Supplementary file 1 [file Data_Sheet_1.pdf]

SUPPORTING INFORMATION

Flagellum is responsible for promoting effects of viable *Escherichia coli* on calcium oxalate crystallization, crystal growth, and crystal aggregation

*Rattiyaporn Kanlaya, Orapan Naruepantawart, and Visith Thongboonkerd**
*(*Correspondence to: thongboonkerd@dr.com (or) vthongbo@yahoo.com)*

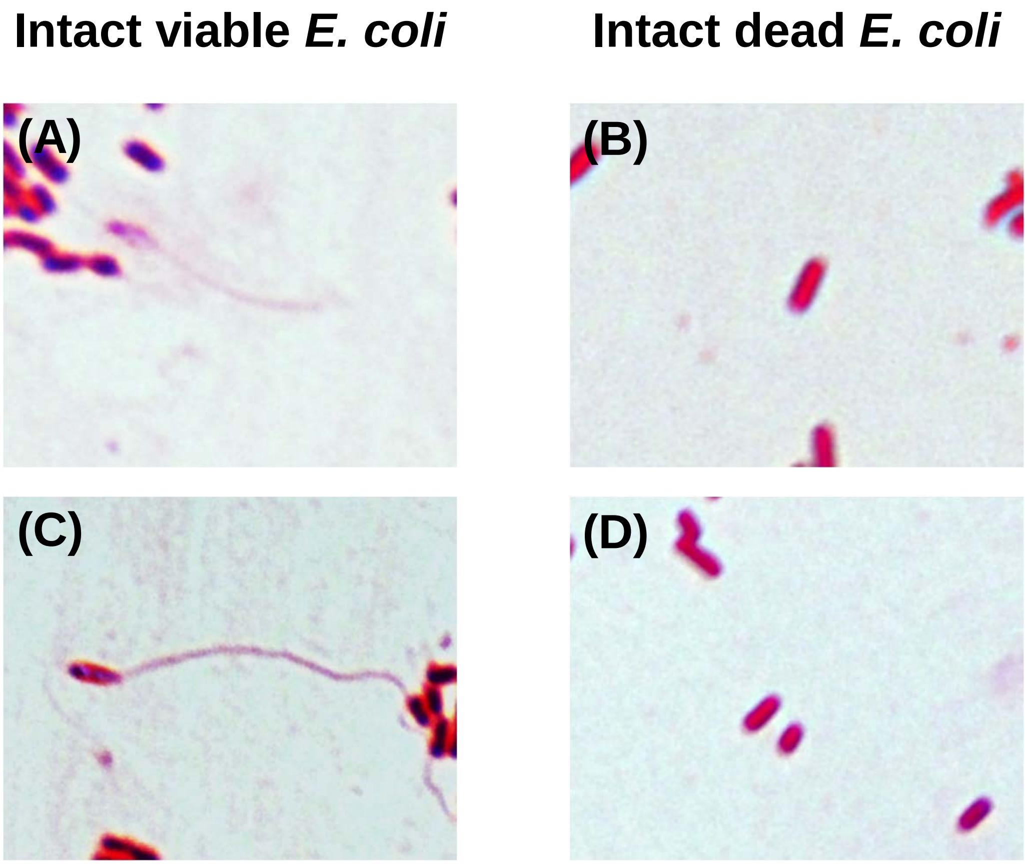

Supplementary Figure S1: Flagellar staining of the intact viable *E. coli* **(A and C)** comparing to the intact dead *E. coli* **(B and D)**. *E. coli* samples were gently dropped onto the water droplet on the glass slides and then air-dried followed by staining using Gray's method. Flagella were observed only in the intact viable *E. coli*, not in the intact dead cells. Original magnification = 1000X.
